# An Evolutionary Explanation for the Perturbation of the Dynamics of Metastatic Tumors Induced by Surgery and Acute Inflammation

**DOI:** 10.3390/cancers3010945

**Published:** 2011-03-02

**Authors:** Alberto Carmona Bayonas

**Affiliations:** Department of Hematology and Medical Oncology, Hospital Morales Meseguer, Murcia, Spain; E-Mail: alberto.carmonabayonas@gmail.com; Tel.: 0034968360900 ext. 4121

**Keywords:** surgery, neoangiogenesis, immune system, evolution, complexity, tumor dormancy

## Abstract

Surgery has contributed to unveil a tumor behavior that is difficult to reconcile with the models of tumorigenesis based on gradualism. The postsurgical patterns of progression include unexpected features such as distant interactions and variable rhythms. The underlying evidence can be summarized as follows: (1) the resection of the primary tumor is able to accelerate the evolution of micrometastasis in early stages, and (2) the outcome is transiently opposed in advanced tumors. The objective of this paper is to give some insight into tumorigenesis and surgery-related effects, by applying the concepts of the evolutionary theory in those tumor behaviors that gompertzian and tissular-centered models are unable to explain. According to this view, tumors are the consequence of natural selection operating at the somatic level, which is the basic mechanism of tumorigenesis, notwithstanding the complementary role of the intrinsic constrictions of complex networks. A tumor is a complicated phenomenon that entails growth, evolution and development simultaneously. So, an evo-devo perspective can explain how and why tumor subclones are able to translate competition from a metabolic level into neoangiogenesis and the immune response. The paper proposes that distant interactions are an extension of the ecological events at the local level. This notion explains the evolutionary basis for tumor dormancy, and warns against the teleological view of tumorigenesis as a process directed towards the maximization of a concrete trait such as aggressiveness.

## 1. An Introduction on Surgeons and Metastasis

If we ignore the biology of the tumors that we are removing, we will certainly not be able to explain the adaptations of the networks that remain. The topic of this paper is the landscape of tumorigenesis that surgery contributed to expose: the distant interactions and variable rhythms. These phenomena were not clearly considered in the classic view; but in recent years, this conviction has crumbled as evidence emerged that surgery could influence the kinetics of the unresected tumors. The most robust data in advanced diseases came from two randomized clinical trials conducted in metastatic kidney cancer, demonstrating a survival benefit from nephrectomy followed by immunotherapy compared to immunotherapy alone [1,2]. Taking both studies together, the adoption of debulking surgery became the standard treatment. With a lower level of evidence, the same advantage seems to occur in other metastatic tumors, such as ovarian cancer [3], germ cell tumors [4], breast [5,6], and colorectal cancer [7]. The reason behind these clinical findings could be due to one of the following: (1) the debulking of a huge mass; (2) the resection of a primary tumor that is qualitatively different from a metastasis; (3) the perturbation of diffuse interaction networks; (4) and, of course, the prevention of local complications.

**Figure 1.**
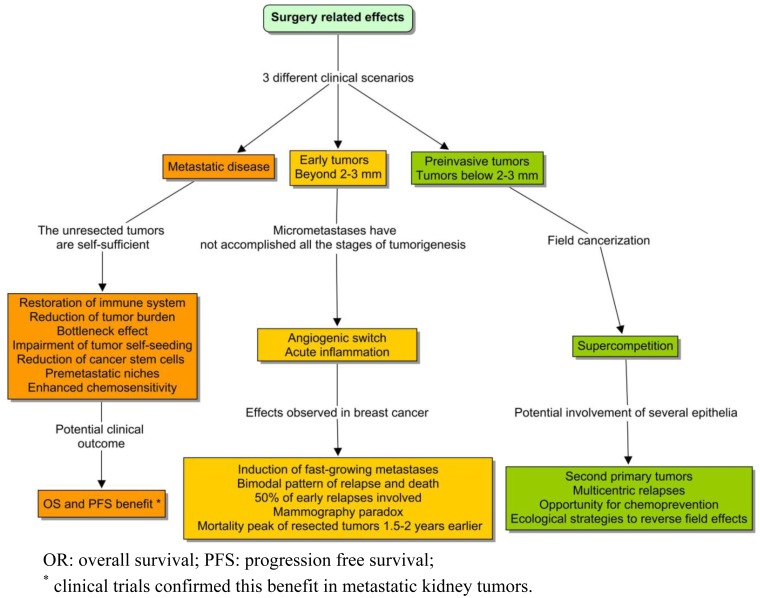
In certain type of tumors, there are three potential outcomes, according to the clinical context. Surprisingly, the work of oncological surgeons argues in favor of “punctuated equilibrium” as a conceptual frame for tumorigenesis.

A substantially different model has been proposed to explain the surgical outcomes observed in early breast cancer [8]. This tumor context is totally different from the previous case, and thereby the surgery effects cannot be compared (Figure 1). A surprising finding was that surgery could induce fast-growing metastasis, and that might explain up to half of the cases of early relapse [9]. This phenomenon appeared to be analogous to the relationship between acute inflammation and tumorigenesis, including the sites of previous trauma [10]. An intriguing empirical fact was also the consistency of the so-called mammography paradox with the pattern of relapse in the series of treated breast cancer. Although most women obtained a benefit from early diagnosis of a breast tumor, the pooled data of mammography trials reflected a paradoxical excess of relapse and death, in a subgroup of young patients with positive lymph nodes. This observation was attributed to a potential iatrogenic effect of screening. The bimodal pattern of recurrence and death in the series of operated tumors was even more intriguing. Those types of curves are expected to follow something similar to a Gaussian distribution. But the Milan series showed a discontinuous function, with a first peak around the second year and a retarded surge around the eighth [11]. The classical notion based on continuous growth was unable to explain this pattern.

In addition, the Bloom database, which reflected the natural history of untreated breast tumors, showed a unimodal surge of death that appeared to delay 1.5 years, with respect to the first peak of relapse in the Milan series [12,13]. In order to explain these facts, some authors introduced the hypothesis of an angiogenic switch due to the decline of systemic inhibitors of angiogenesis following surgery [14]. That mechanism could explain a proportion of early relapses, but the late ones still appeared to be responding to different underlying phenomena. Independently of the reason, it was clear that if any of these clinical effects were confirmed, the hypothesis of unconnected tumors of continuous growth could not be held. And in the future, the aim of specific interventions could be to maintain metastatic cells asleep throughout tissular cataclysms.

## 2. How to Unleash Cell Competition in a Multicellular Organism

A tumor is a complex entity that deserves a special type of explanation. In the following sections, I shall argue that this explanation is natural selection. This view matches all the biological observations into a coherent paradigm, which includes an uncomplicated mechanism to explain the driving force of tumorigenesis. Somatic evolution clarifies the origin of distant interactions, and the source of variable rhythms of progression. Cancer is usually presented as a genetic disease of the cell cycle, caused by the accumulation of somatic mutations.

The evo-devo description has incorporated this vision, but also entails a broader range of observable facts including the co-option of development genes, the influences from stromal factors, and the effects of cell evolution on tumor-host interactions [15]. Notably, this depiction implies that the same mechanisms of evolutionary change (inheritance, variation and reproduction) [16], which explain the appearance of biological complexity persist within multicellular entities [17,18]. The cancer cell insensitivity to anti-growth signals is a key component of the depiction, because it gradually disengages tumors from the regulatory networks of multicellularity. The presence of genetic instability confers unique traits to the whole process and also makes it atypical from the point of view of normal development or tissue remodeling. In particular, the accumulation of DNA damage entails three main consequences: (1) the abnormal activation of many parallel programs; (2) the appearance of cell heterogeneity, and (3) the emerging of ecological processes. It is shocking that Darwin's fight for survival is essentially what makes tumors so hard to treat effectively.

Basically, genetic instability is the main source of genetic variation that cell evolution requires. Tumor cells are capable of accumulating more than 11,000 different mutations with respect to healthy cells and still continue to thrive [19]. These heterogeneous clusters can do very well because they do not precisely follow many of the genetic toolkits of multicellularity. As a result, phenotypic diversity naturally organizes the tumors into subpopulations with respect to the hallmarks of cancer [20]. In the next step, ecological interactions between cells emerge from decreasing resources and other pressures. Cell diversity is then correlated to the collective behavior and prognosis of tumors [21]. It is also interesting to comment that all the different subclones are genetically connected, and thereby cell genealogy can be represented by a phylogenetic tree with a unique root [22].

## 3. Competition in a Tumor Smaller than 2-3 mm

Despite the evolutive regulation of the cell-cycle, multicellularity did not entirely get rid of the competitive nature of eukaryotic cells [23]. Genetic diversity provided the source for ongoing local competition. Thereby, the acquisition of selective advantages becomes the cornerstone of tumorigenesis. Cell competition was first observed in *Drosophila melanogaster*, when technical improvements allowed creating patches of mutant cells surrounded by wild-type tissues. It was shown that heterozygous mutations in ribosomal genes (Minute genes) produced almost normal phenotypes in homotypic tissues. However, these mutant populations exhibited a holdup in growth and were purged when confronted with wild-type cells in mosaics [24].

It was also observed that other genetic aberrations could induce a state of supercompetition, in which a “strong clone” was able to eliminate the surrounding wild-type cells [25]. The overexpression of *C-myc* was shown to induce supercompetition in early stages of carcinogenesis, and actually it appears to be one of the links among tumor dormancy, competition, angiogenesis and immunology. Its expression prevents the transcription of angiogenesis inhibitors such as thrombospondin, whereas its inactivation was shown to lead to tumor dormancy. *C-myc* is also able to recruit inflammatory cells that cooperate with angiogenesis [26,27]. The expansion of mutated cells with stem cell properties is a striking reminiscence of the well-known process of field cancerization. This notion encompasses the existence of patches of genetically aberrant cells in histologically normal epithelia. It should be an important opportunity for chemoprevention to unveil the role of inflammation, recurrent infections and other environmental cues, on the emergence of second primary tumors from the field [28].

Other stromal factors provide the selective pressure that is required for a clonal expansion of mutant alleles. This is especially striking in the natural selection of the common pathways of proliferation and metabolism. The activation of the *KRAS/MAPK* signaling pathway is known to induce cell proliferation, but the precise ecological mechanisms involved in the expansion of their mutant alleles have not been completely elucidated. Yun *et al.* have recently proved that hypoglycemia is the driving force that raises the frequency of *KRAS* and *BRAF* mutant alleles during early tumorigenesis, by means of ecological selection in the primary tumor [29]. The rationale involves the vulnerability of the wild-type cells to low glucose levels. The mutational activation of the *MAPK* signaling pathway upregulates the glucose transporter *GLUT1*, with an increase in glucose uptake and anaerobic glycolysis. This trait provides tumor cells with a steady proliferation advantage in a background of scarcity, finally leading to the expansion of the mutant allele in the population. Interestingly, the acquired metabolic changes are stable, which in fact constitutes the main reason why PET scans are efficient to detect preinvasive polyps. Interestingly, *KRAS* mutations have also been involved in field cancerization [30], so they can be found in non-neoplastic tissues.

There is a similar basis for the so-called Warburg effect, the continuous and self-correcting process by which tumor cells obtain a selective advantage by virtue of a switch to a less efficient metabolism. In fact, the use of anaerobic glycolysis instead of the tricarboxylic acid cycle (TCA) provides less *ATP* per glucose molecule, but maximizes the diversion of glucose and glutamine to the synthesis of structural molecules.

**Figure 2.**
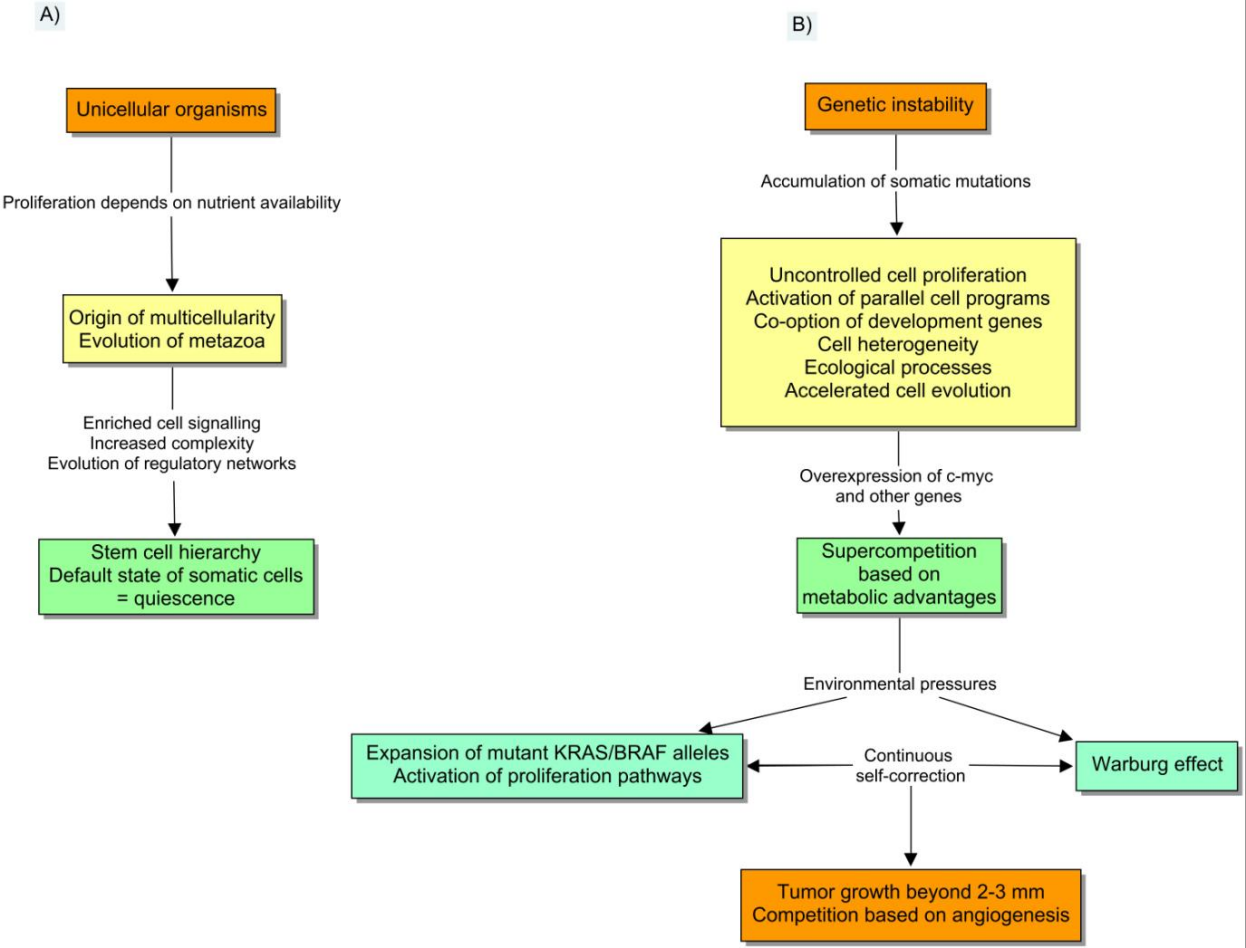
How to “construct and deconstruct” a multicellular organism. (a) The regulation of the asymmetric division of stem cells is an evolutionary trade-off between terminal differentiation and proliferation. The origin of multicellularity entailed large increases in signal transduction pathways, to guarantee and regulate these hierarchies. So, unregulated cell proliferation constitutes a pathological state. (b) Genetic instability is linked to the appearance of ecological interactions, and it makes tumorigenesis different from other processes such as embryological development, and other emergent phenomena of gene regulatory networks. Notice that the reverse process of “metazoan evolution” is cancer.

Interestingly, one of these mechanisms of diversion is the expression of *pyruvate kinase M2 isoform*, a fetal isoenzyme that is regulated by phosphorylation when proliferation signaling pathways are active [31]. This regulation reduces the flux of pyruvate into mitochondria, slowing the rate of the TCA cycle before the exposure to hypoxic conditions. The emission of large amounts of lactate as a result of anaerobic glycolysis reduces the microenvironmental pH with further selective consequences. In particular, the activation of *HIF-1α* induces several cell programs including the beginning of a new type of competition based on neoangiogenesis (Figure 2).

## 4. Ecological Events in Tumors Larger than 2-3 mm

It is well known that when solid tumors grow beyond that size, neoangiogenesis is inevitably the main limiting factor to sustain further growth. Here, I will discuss some interesting clues suggesting a complex engagement of neoangiogenesis and the complementary processes of invasion and metastasis. The heterogeneity of angiogenesis among tumor subclones is a key factor to consider at this phase of tumorigenesis [32-34]. The endogenous inhibitors of angiogenesis are regarded as critical regulators of the pathological angiogenic switch [35]. Several of such molecules have been found, of which many are fragments of extracellular matrix proteins [36]. So they are collectively induced by processes in which the activated stroma is involved, such as inflammation, growth factors, the cascade of proteases and other mechanisms. The physiological role of the angiogenic inhibitors is to provide a tight and delicate regulation of angiogenesis when it is locally triggered.

The preclinical studies of antiangiogenic drugs give an insight into the molecular strategies of cells to avoid the constriction due to angiogenic inhibitors. Although these data are not definitive, they supply convincing arguments that ecological processes among subclones are taking place at this scale. A compelling observation is that an adaptive upregulation of pro-angiogenic pathways is involved in the development of an evasive resistance to angiogenesis inhibitors. Casanovas *et al.* [37] studied the blockade of *VEGFR* in a mouse model of carcinogenesis, and observed that after a transient response, the tumor always progressed. The authors were able to prove that evasive resistance was the result of the increased transcription of several mRNAs belonging to alternative pro-angiogenic factors, such as *FGF-1*, *ephrins* and *angiopoietin-1*. Other reports have shown that the ectopic expression of matrix-derived antiangiogenic factors, such as *endostatin*, *tumstatin* and *thrombospondin-1* suppresses tumor progression transiently [38]. However, the subsequent activation of different proangiogenic pathways, including the up-regulation of *VEGF*, *PDGF* and *FGF*, allows the tumor cells to bypass and escape from the restriction. Interestingly, the physiological response to hypoxia, which naturally includes the overexpression of *HIF-1α*, and the induction of *VEGF* and other mediators, is convincingly related to a natural resistance to antiangiogenic factors.

## 5. Invasion and Metastasis Include Distant Interactions

I will defend here that during the course of tumorigenesis, competition is extended to a superior and more sophisticated level, with mediators such as angiogenesis inhibitors and immune response modifiers. Although in the beginning, tumorigenesis can be depicted as a contest among tumor subclones that strive for local resources, during the progression of metastasis, the ecological interactions tend to approximate to distant antagonism exerted by soluble factors.

Epithelial to mesenchymal transition (EMT) turns out to be the most critical cell program to acquire an invasive and mobile phenotype. EMT is the set of events that collectively induce epithelial cells to get hold of mesenchymal traits, which include the loss of intercellular bonds, and the capacity to migrate and infiltrate. Although there are many cues capable of unleashing EMTs, they can be classified into three major types according to the context and the nature of the biological result [39]. Type 1 EMT is basically a program of embryological development. Therefore, it is highly regulated by the molecular toolkits of embryogenesis such as *Nodal*, *Wnt*, *TGF-β* and several transcription factors. In contrast, type 2 EMT is linked to wound healing and tissue remodeling. Here, the course of action is also modulated by extracellular stromal signals. Otherwise, type 3 EMT is basically a collection of events associated with tumor invasiveness including metastasis. It is driven by the cooption of the same regulatory circuitry than in type 1 EMT, and also suffers the same stromal interactions attributed to type 2. The main dissimilarity that confers its idiosyncrasy to type 3 EMT is basically the emergence of genetic instability and the abnormal sensitivity to exogenous signals.

Hypoxia is engaged with two important cell survival programs: angiogenesis and EMT. If cells can bring oxygen to the site by means of neoangiogenesis, they remain there, otherwise, there is an ongoing selective pressure to activate the alternative program, to move somewhere else. So, if a cell program fails, they have the other one, that actually remain intricately coupled. For example, both *HIF-1α* and VEGFR1 are critical inducers of angiogenesis, but their expression also mediates EMT [40,41]. To understand this regulation, we should be aware of two important subtleties: (1) proliferation and EMT are sometimes strikingly uncoupled, and (2) EMT activates matrix-metalloproteinases that cleaves extracellular-matrix components, such as type XVIII and IV *collagen*, to produce matrix-derived angiogenesis inhibitors [42]. I will to try to discuss the biological value of these observations.

**Figure 3.**
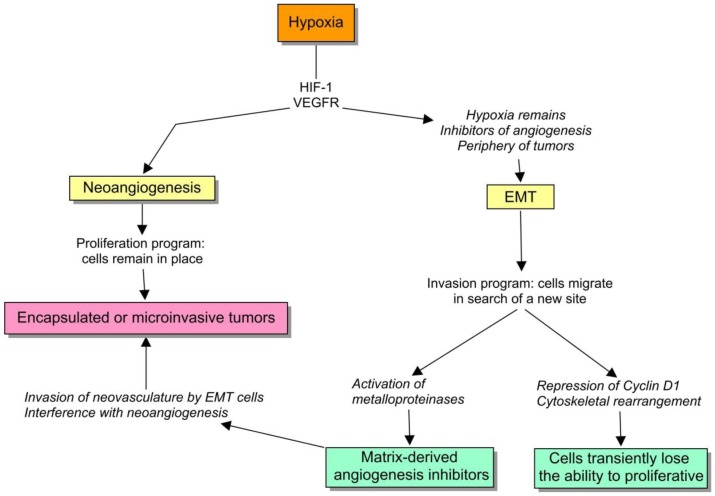
Environmental pressures contribute to make the choice between neoangiogenesis and EMT. The balance between both processes is a suitable target for natural selection, operating at the somatic level.

First, the transcription factors involved in EMT, such as *slug*, *snail* and *SIP1* are negative regulators of proliferation via the downregulation of *cyclin D1* expression, and other inducers of the cell cycle [43-45]. It actually makes sense because the radical cytoskeletal rearrangement that is required during cell movement is not really compatible with mitosis. Therefore, the cells undergoing EMT lose their proliferative capacity, and thereby the balance between EMT and angiogenesis can be a suitable target for natural selection (Figure 3).

Notably, this fact is also responsible for the emerging of biphasic forms of life in unicellular eukaryotic organisms and contributed to the evolution of multicellularity, in which it still may represent a critical constriction (see the ecological problem in the evolution of *Volvox* [46]).

Brabletz *et al.* [47] compared the central and the peripheral zones of primary tumors and metastasis, with regard to the expression of epithelial markers and Ki-67. They showed that the core of the tumors mainly consists of well-differentiated glands, with normal epithelial markers and high expression of Ki-67. Therefore, this part of the tumor is forming neovasculature and proliferating very actively. But strikingly, in the front of invasion, the scattered cells have mesenchymal markers and do not express Ki-67. So those cells are invading and emigrating, but not proliferating. In fact, they have disengaged both processes.

**Figure 4.**
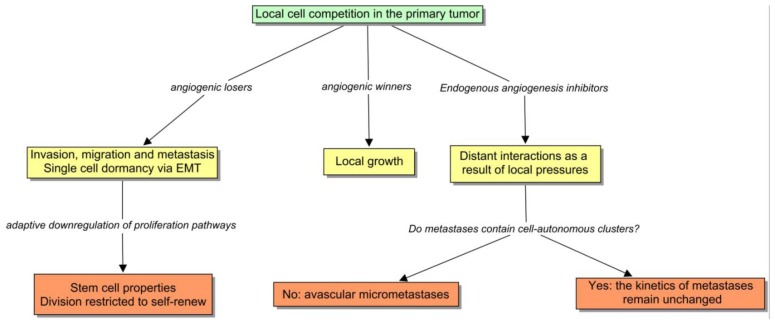
The competition between tumor subclones for the resources provided by neoangiogenesis, classifies cells in angiogenic winners and losers. Distant interactions are the result of the extension of local pressures.

This is a compelling clue that suggests that EMT is the preferred cell program in certain conditions such as: (1) when neangiogenesis is not sufficient; (2) or when it is strongly blocked by endogenous inhibitors or by drugs; (3) or when the tumor vasculature is harshly disrupted [48], and also (4) as Brabletz *et al.* proved, in the periphery of the tumor, where cells receive stromal cues directly. In these situations, cancer cells seek the alternative mechanism to angiogenesis, which involves invasion [49,50]. Interestingly, this behavior has been checked in the clinical setting, in which antiangiogenics appear to promote multicentric patterns of progression [51]. If we now return to the ecological paradigm, it is appealing to envision a model in which the subclones can be divided into angiogenic winners and losers (Figure 4). The former ones would tend to remain encapsulated or microinvasive, and the losers would form large fronts of invasion that constitute the basis of metastasis. Paez-Ribes has recently demonstrated that *VEGFR2* blockade is linked to a higher dissemination and tendency to metastasize in a transgenic mouse model [52]. Notably, in this experience, lymph-node metastases were 4-fold higher in treated animals than in controls, and liver metastases were 2-fold higher, and very interestingly, the invasive cells were also able to invade the adjacent microvasculature as expected. The development of evasive resistance to drugs may involve the same mechanism. Elbos *et al.* [53] have reported an accelerated metastatic tumor growth in mice models treated with the *VEGFR/PDGFR* inhibitor sunitinib, even though this treatment is effective *in vitro*. This observation is outstandingly reminiscent of some recent clinical research results. Both bevacizumab and cetuximab are successful in treating metastatic colorectal cancer. Taking into account that *VEGF* and *EGF* pathways are connected, the dual inhibition was supposed to be an attractive strategy. In fact, preclinical studies had shown the synergy between anti-EGFR and antiangiogenic therapy. Two recent randomized clinical trials notably apply this notion, the PACCE and Cairo-2 trials, failed to confirm this preclinical assumption. In fact, the dual inhibition yielded a poorer result, worsening the PFS of the whole population [54,55].

In aggregate, these observations point out that tissue effects always need to be considered to predict the outcomes. In particular, both the activation and suppression of neangiogenesis, depending on the context, can elicit pro-invasive behavior. These peculiarities of EMT could explain why tumor dormancy is a final adaptation of single cells, and why metastasis progression genes are selected in primary tumors [56]. Next, I will discuss the relevant implications of how EMT is engaged with the induction of matrix-derived inhibitors of angiogenesis.

## 6. Surgery, EMT and Inhibitors of Angiogenesis

Invasion is coupled with the activation of the stromal network of metalloproteinases (MMP) that contributes to cleave the components of the ECM, mainly *type XVIII and IV collagen*, into fragments that work as endogenous inhibitors of angiogenesis. There are many of these protein pieces such as endostatin, tumstatin, arresten or canstatin that can mount a counter-regulatory response [57,58]. The effect of these molecules imposes a selective pressure to make the choice between angiogenesis and EMT. A microinvasive tumor is expected to perturb the extracellular matrix less than an overtly invasive lesion. Besides, too much EMT can potentially interfere with angiogenesis in the core of the tumor. So this negative feedback can supply the basis for a balance between both cell programs. Some angiogenesis inhibitors are expected to work locally, but interestingly, humans also appear to be in possession of a systemic antiangiogenic toolkit. Occasionally they are detectable in plasma, where it can be tricky to interpret what they are doing. However, one of their functions can reveal the solution to the silent cancer conundrum. Some epidemiologic reports have found an incredibly elevated rate of tumors in the general population, but most of them will not ever progress [59,60]. An intriguing observation reported in patients with Down syndrome can shed light on this paradox. Breast cancer incidence is very low in women with the trisomy of chromosome 21. One of the possible explanations is that the chromosomal aberration provides at least 50% higher endostatin levels [61] than the normal ploidy. Curiously, it does not result in an alteration of wound healing or regeneration [62]. In view of these observations, I suppose that the gauge of our angiogenic network can be an anachronistic adaptation. The picture is similar in patients with tumors who have been operated on early, in which dormant micrometastases in the bone marrow are prevalent, but only half of them tend to progress to fast-growing diseases [63].

During the course of tumorigenesis, local pro-angiogenic factors prevail over stromal and systemic inhibitors. I propose that, from the point of view of the tumor subclones, the ecological dynamics can be interpreted as follows: (1) a tumor patch containing an effective angiogenic phenotype tends to displace the rest of the clones; (2) the local expansion of the prevailing cells increases the selective pressure to the neighbors, primarily as a contest for neighboring resources, and later by inducing a stromal release of angiogenesis inhibitors; (3) when the dominant subclone expands to a critical level, the overflowing inhibitors exert local effects on the systemic setting [64]. One of the surprising interpretations of this model, taking into account the phenotype that emerges during EMT, can be that metastasis are the adaptation of a “population of angiogenic losers” impeded to proliferate locally. This argument can be considered an extension of the Folkman hypothesis with regard to the inhibition of metastasis by growing primary tumors. I think the clues are quite compelling, thereby it would warn against a teleological view of tumorigenesis as an evolutionary process directed towards the maximization of a concrete trait such as aggressiveness. Occasionally, it could also include long periods of dormancy. In fact, I shall argue that the genetic profiling of dormant tumors is compatible with this ecological view (see the next section).

If it is tricky to interpret the meaning of blood angiogenic inhibitors in healthy individuals, it is even more difficult in patients with cancer. The growth of the primary tumors makes use of these molecules to slow down the kinetics of metastases [65,66]. In the same manner, it has been proven that long-term dormancy can be induced by physiological concentrations of circulating angiogenesis inhibitors, even after the removal of the primary tumors [67,68]. But according to the origin of the molecules, they may reflect the burden and aggressiveness of tumors, without a significant change in the kinetics of metastases. In these cases, the systemic angiogenesis inhibition is clearly exceeded by the local pro-angiogenic products [69,70]. Besides, preoperative endostatin is elevated in patients with cancer in comparison with healthy controls, but this variable is not associated with an indolent behavior as expected, but with poor prognosis and large tumor burden [71,72]. Here, endostatin behaves as an innocent bystander rather than a driving force. In the same manner, a significant increase of the endostatin level after surgery is correlated with poor outcome in patients with metastatic kidney cancer [73]. Moreover, high endostatin levels also predict a lower time to progression in patients with breast cancer treated with aromatase inhibitors [74]. However, it is also necessary to take into account that blood endostatin levels may not represent what is happening at the local level.

Tumor invasion and EMT are held responsible for the preoperative levels of endostatin in some reports [75], but it can also be produced by the activation of metalloproteinases during collagen remodeling and wound healing. Therefore, surgery can show a biphasic pattern of endostatin and other inhibitors, with an early drop and a posterior increase. Wu *et al.* [76] showed that breast surgery implied two pathways of angiogenesis induction, as they reported a local 9-fold increase of *VEGF*, but a transient 30% dip of endostatin during the first four days following resection. But the drop associated to tumor debulking is expected to be transient. Some studies have reported an increase of postoperative endostatin several weeks after surgery, so the source cannot be the primary tumor but the surgical wound [77]. The main question is actually whether this short-lived burst of proangiogenic factors is necessary to explain the discrepancy between Milan and Bloom series, and the so-called mammography paradox. In fact, it appears that it is [78,79], but a mechanism to amplify the effect is needed, and, in my opinion, it supports the view of the cell-centered models of tumorigenesis (see below).

## 7. Tumor Dormancy and Fast Growing Metastases

Efforts to try and understand the remote causes of the irregular rhythms of tumor progression contemplate two singular components: (1) the angiogenic switch of avascular micrometastastes, and (2) the arousal from single cell dormancy. A seeding with silent micrometastases is a common trait of apparently localized tumors [63]. Although some of them may demonstrate a high proliferative activity, in absence of neoangiogenesis they are condemned to balance every cell division with apoptosis. The arousal of this type of micrometastases can be influenced by a punctual perturbation in the network of distant interactions, involving some type of tissular catastrophe, such as surgery, traumatisms, or acute inflammation.

By contrast, single cell dormancy is the result of an intrinsic downregulation of proliferation and angiogenesis. For example, the activation of p38 over ERK induces single cell dormancy in some models [80,81]. Both mechanisms may occur together at the same time. Although this is something far from being conclusive, I will explain that they are not independent phenomena but share a common origin, which derives from the local selective pressure. I have also hypothesized that dormant cells come, probably via EMT, from a population of angiogenic losers, hindered to proliferate locally. I will expand the topic of “adaptive cell quiescence” here.

Although the ability to form rapidly-growing macrometastases from dormant foci is probably the least well understood phase of tumorigenesis, at least four critical requirements can be recognized at the cell scale: (1) the necessity to revert EMT; (2) the upregulation of proangiogenic pathways to counteract the effect of systemic inhibitors; (3) the activation of *PI3K/Akt* and other signaling pathways linked with proliferation, metabolism and survival, and (4) the adaptation to stromal signals.

The induction of mesenchymal to epithelial reverting transition (MErT) is considered a prerequisite to the reactivation of proliferation pathways [82]. MErT is a fascinating topic to explore. The adherens junctions linked to E-cadherin contribute to mount a biological barrier that can prevent some cell damage, including the response of the immune system, and also provide new interactions with stromal receptors and other mechanisms. So this connects with the speculation that only epithelia are able to provide a critical density of homotypic cells to sustain collective effects, such as angiogenesis, shield from the environment, *etc.* This is an outstanding reminiscence of quorum sensing regulation of cell behavior in nature [83]. In fact, single epithelial cells have an interesting problem: Precisely to be isolated in the middle of an ectopic parenchyma. In the beginning, it obliges them to bind to non-neoplastic epithelial cells [84], and to depend on their influence to survive. So they are less autonomous than they should be according to their own traits.

But there is a second reason for MErT. It is well-known that *E-cadherin* binding retains *β-catenin*, what prevents its nuclear localization and association to the *Tcf/Lef complex* in order to activate transcription programs. So this is one of the links between a transcriptome change and cell proliferation. In the following paragraphs, I will focus on this important issue. The transcriptional analysis of dormant tumors shows that critical components of the proliferation networks, such as the *PI3K/Akt* axis, tend to be downregulated [85]. The *PI3K/Akt* pathway is a central mechanism of EMT, since it upregulates some transcription repressors such as *Snail*. In dormant cells, the repression of *PI3K/Akt* is able to induce the re-expression of epithelial markers [86]. So the default state of these migrant cells tends to induce MErT, as soon as the stromal cues from the primary tumor have gone. There is an interesting feedback loop here, because once *E-cadherin* binding is restored, it is able to activate the *MAPK* and *PI3K/Akt* cascades that mediate cell proliferation and survival [87].

But this depiction is even more complicated. Almog *et al.* [85] have analyzed the expression pattern of genes that predominate in dormant and fast growing tumors. So they have been able to describe which genetic mechanisms are important in the transition between both scenarios. Intriguingly, they have found that thrombospondin, angiomotin, tropomyosin, *TGF-β2* and *IGFBP-5* are upregulated in dormant tumors. In fact, one of the roles of *TGF-β2* can be to sustain EMT, once the influence of the stromal signals from the primary tumor has disappeared [88]. Otherwise, in the transition to fast-growing tumors, the upregulation of several pathways related to neoangiogenesis and proliferation, such as *EGFR*, *IGF* and *Notch* pathways, can be important events at the molecular level. It is very important to elucidate the driving forces of the transcriptional changes at this transition, because they will probably constitute an attractive opportunity for cancer adjuvant therapy.

Although the report by Almog *et al.* [85]. is quite compelling, and it appears to favor an intrinsic and cell-centered behavior, tumor dormancy can also be a trait influenced by the colonized tissue. It is well known that some clusters of metastatic cells persist unchanged for prolonged periods of time, due to the influence of the extracellular matrix [89,90]. The problem gets more complicated when the selective pressures of treatments are taken into account. The sensitivity to hormone therapy in breast cancer is strongly related to tumor dormancy. Chemotherapy preferably destroys the proliferating cells, and selects a quiescent phenotype. But even considering these scenarios seriously, it is still compelling to envision a model in which the dormant phenotype directly emerges within the primary tumor, as some subclones are hindered to propagate locally, so they develop the ability to invade and seed distant tissues. Although they are endowed with great cell plasticity, this dormant phenotype remains stable for a while, at least until new stromal influences arise, and new mutations and epigenetic changes are recruited [85,91].

Actually, epithelial plasticity involving cancer can be something faster and more sophisticated than previously thought. Dykxhoorn *et al.* [92] have recently demonstrated that the breast cell lines with an elevated expression of *miR-200* surprisingly produce more macroscopic metastases when they are injected into mice, in comparison with the cell lines showing a lower expression. This observation is very interesting because it was exactly the opposite result to that expected. The *miR-200* family is thought to promote MErT by inhibiting *Zeb2*, which thereby upregulates *E-cadherin*. So *miR-200* was theoretically expected to reduce the capacity to invade, and therefore to inhibit metastasis, not to promote them. This result supports the view that tumor colonization is enhanced by MErT, but the epithelial phenotype of cells did not predict the metastatic outcome. The situation is not so tricky if we understand that epithelial plasticity may endow cancer cells with the ability to revert their phenotypes very rapidly *in vivo*. This is likely a gradual process with intermediate forms, and thereby it is also possible that a complete EMT with the full set of traits is not always required to produce metastasis. Gavert *et al.* [135] have proved that some metastasis do not require changes in EMT. The expression of the neural cell adhesion molecule L1 provides cells with the ability to invade and produce metastasis, without losing their epithelial markers. Therefore, a trade-off of epithelial and mesenchymal traits could optimize the adaptation in both the primary tumor and distant tissues.

Here, there is an underlying connection with the tumor stem cell theory. It is well-known that EMT produces cells with stem-cell properties [93], involving the capacities of self-renew and differentiation. They have been found in most human tumors. Al-Hajj *et al.* concretely proved the existence of breast cancer stem cells [94]. They showed that only a tumor subpopulation with the phenotype CD44+CD24- had the capacity to transplant breast cancer in animal models, which is precisaly the phenotype provided by the EMT process. So when the tumor nests progress, this subpopulation is able to regenerate the complexity of the original tumors, and to produce new metastases. In this scenario, it is important to understand that single dormant cells are still capable to self-renew.

**Figure 5.**
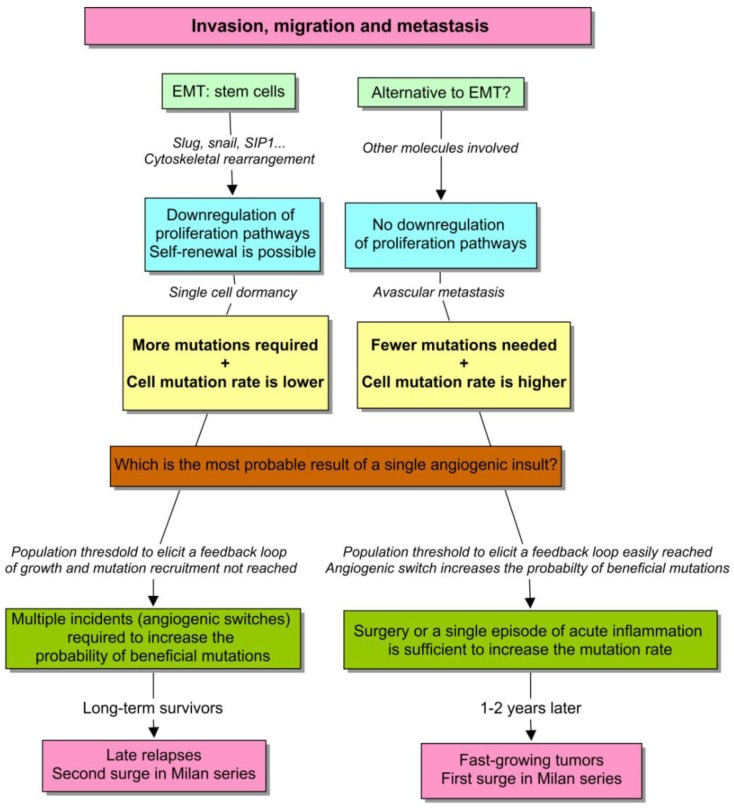
Amplifier feedbacks in the evolution of dormant tumors. The discrete behavior in the Milan series can be explained by a positive feedback loop that amplifies the effect of a short-lived angiogenic insult. Any population growth, even though it is small, implies that the mutation rate increases to a certain extent. Therefore, the mutational upregulation of angiogenesis and proliferation pathways is more likely to develop. This upregulation closes the circle and allows a more rapid recruitment of other metastasis progression genes. However, the population increments as responses to the same angiogenic stimulus are unequal, and depend on the cell ability to proliferate during the burst of angiogenesis.

With this in mind, we should envision a model to describe: (1) how a discrete macroscopic behavior emerges from a continuous cell phenotype, and (2) why an unsustained burst of angiogenesis is able to perturb the kinetics of micrometastases in some cases but not in others. First, a continuous spectrum of cells with different cancer traits is probably the most suitable and realistic scenario. However, to clarify the emergence of discreteness, I will consider a natural distinction that appears to be evident (see Figure 5). There are epithelial cells that proliferate actively, and mesenchymal cells with stem-cell properties that only self-renew. Both need to acquire new mutational equipment to acclimatize to the metastatic site. But in the former example, the number of required mutations is much lower, in comparison with the latter. This premise appears to make sense when considering the report by Almog *et al.* [85]. Notice that in both cases, how long it takes to recruit a new mutation and therefore, to produce a fast-growing tumor essentially depends on the effective population size of the dormant tumor, and the probability of “beneficial” mutations in single cells. So everything that increases these parameters is likely going to accelerate the process of tumorigenesis.

However, in tumors of the same size, a higher proliferation rate also yields more mutations. So the population growth depends on cell-specific and environmental factors. Besides, considering the same perturbation, a short and unsustained stimulus is capable of increasing the population faster in cells with an active program of proliferation, with respect to quiescent cells. So we have the depiction of an unstable phenotype, affected by a positive feedback loop that amplifies the effect of a punctual perturbation, such as surgery. The discontinuity in the Milan series, and particularly the first surge, can be explained on this basis.

The second phenotype is much more stable instead. Since, proliferation is restricted to self-renewal, and stem cells have an asymmetric division that protects them from mutations, the effective population size is less affected by an isolated episode of angiogenesis. The progression here is instead the result of multiple, small and unpredictable incidents throughout the life of the patient, such as traumatisms, infections, chronic inflammations and others [10]. These type of micrometastases have the double problem of needing more mutations, when it takes longer to recruit each one of them. In this scenario, an amplifier feedback is more difficult to develop, although it can inexorably occur in more advanced stages. This behavior can explain both the second surge in Milan series and the strikingly late relapses of breast cancer.

This evolutionary approach can be simplistic in some points. Although the process is apparently triggered by both environmental change/stress and stochastic mutations, an initial perturbation of the epigenetic regulation of the tumor cells is also feasible. In nature, an intriguing hypothesis proposes an epigenetic basis for punctuated equilibrium based on the internal genetics of organisms as a complement to an environmental forcing [95].

## 8. The Tumor has Completed All the Stages of Tumorigenesis

Tumorigenesis is a multistep process. When tumors have completed all the stages, they are almost autonomous entities. However, that self-sufficiency does not imply either the finish of distant interactions between tumor sites, nor the absence of a significant perturbation after surgery. But it is obvious that the context is going to be completely different to what was discussed so far.

The pathophysiological background of these interactions can be diverse, since this scenario is not homogeneous. There are two main situations, the resection of synchronic or metachronic metastases, and the resection of primary tumors in metastatic patients.

First, there are a number of studies that report the outcome after the resection of oligometastases, mainly of colorectal cancer. The variables associated with a poor prognosis in this setting are: a node-positive primary tumor, a short disease-free interval, the presence of synchronic rather than metachronic metastases, the number, size and distribution of lesions and the level of CEA [96,97]. Metasectomy is the preferred option, because overall five year survival rate with an R0 resection is around 37% and 64% in the groups with good prognosis [98]. In aggregate, tumors with more capacity of invasion tends to be more aggressive. The disease-free interval in this scenario can be a marker of the average difficulty to complete tumorigenesis.

Second, the resection of the primary tumor has been linked to a better prognosis in several metastatic diseases. As I have mentioned, two randomized clinical trials conducted in metastatic kidney cancer demonstrated a survival benefit from nephrectomy followed by immunotherapy compared to immunotherapy alone [1,2]. But this benefit can also occur in other types of tumors such as melanoma, gastric, breast, colorectal, and ovarian cancer [3,5-7,99,100]. So the question is which type of networks are perturbed on each occasion and which is the origin of such types of interactions.

### 8.1. The Role of the Immune System in Advanced Diseases

The restoration of the immune system has been involved to explain the benefit of surgery in the metastatic setting. First, Danna *et al.* [101] described a mouse model in which the removal of a primary tumor was able to restore the antibody and cell-mediated response, even in mice with a remaining metastatic disease. Then, some investigations also showed that human primary tumors were the main sources of immunosuppressors, and that was correlated with the clinical behavior. Particularly, the aberrant expression of some molecules can induce T-lymphocyte dysfunction and apoptosis, via the suppression *NFkB* pathway [102]. These observations were supported by the reduction in circulating immunosuppressors after nephrectomy, which was probably responsible for the clinical benefit [103]. So those reports suggested that cancer is associated with a profound immunosuppression, but the resection of the primary tumor is able to reverse this situation even in presence of clinical metastases.

Interestingly, the origin of immunosuppression can also be the extension of the ecological events at the local level. Multicellular organisms developed a program to eradicate tumor cells called “immunosurveillance” [104,105]. The infiltration of T-lymphocytes in tumors is an early event of tumorigenesis, whose extent is correlated with a favorable prognosis [106]. Tumor-specific antibodies also predict the outcome in advanced-stage diseases [107]. But this pressure selects cells with attenuated immunogenicity, which are adapted to survive in an immunocompetent host [108]. Consequently, the progression of primary tumors is associated with traits such as antigen-loss, reduced immunogenicity, down-regulation of the major histocompatibility complex (MHC) and immunosuppression [109,110].

Moreover, cancer is also able to educate the immune cells to promote an aggressive behavior [111,112]. The process is called immunosculpting. The molecules of the inflammatory cascade (*TGF-β*, *COX*…) are used to recruit fibroblasts that in turn synthesize soluble factors, which promote survival, angiogenesis, and contribute to EMT, with a clear role in invasion and metastasis [113]. This process can also be a source of ecological interactions, and it has been described in terms of coevolution, in which positive feedback loops benefit both tumor cells and macrophages [114].

These strategies comprise substantial intratumoral heterogeneity, and probably constitute one of the traits that differentiate primary tumors from metastases. Natural selection operates on immunogenicity variations, so it is feasible for a subclone to elicit a response against an adversary, as a mechanism of competition [115,116]. It has been proved that the immune cells can trigger tumor dormancy and even tumor cell disappearance [117,118]. In fact, immunity can be effective in some early and homogeneous tumors [119,120]. However, in most metastatic cancers, the overflowing immunosuppressors transfer the local effects to the systemic setting. In this stage, the immune system becomes exhausted, but debulking surgery can still be effective to alleviate immunosuppression.

### 8.2. Surgery Reduces the Tumor Burden, Genetic Diversity and Metastatic Seeding

The resection of primary tumors can reduce the global tumor burden. Since the number of metastatic sites and the total account of cells is linked with the tumor prognosis, a local resection could change the behavior of the remaining disease. Interestingly, Fehm *et al.* [121] proved that the pattern of chromosomal aberrations in circulation tumor cells (CTCs) agrees with the karyotypes in subclones belonging to the primary tumor, so this is a proof that CTCs really originate there. In my opinion, it could be interesting to explore if the primary tumors, as the cradles of cancer, harbor the whole miscellany of genetic diversity of the disease, as it was shown at different scales in other evolutionary phenomena [70]. Since a large account of CTCs is associated with enhanced tumor progression in some studies [122], surgery may also improve the outcome by means of reducing the amount of CTCs.

Population heterogeneity is also an independent risk factor for poor prognosis [123]. The reduction of the tumor burden can be associated with a decrease in the level of heterogeneity. In fact, this would be the case if a primary tumor was different from a metastasis, and thereby the effect of removing any of them could be distinct. A bottleneck effect in genetic diversity is a possible result of the resection, and it probably correlates with a better outcome.

In fact, a postsurgical genetic bottleneck can be implicated in the development of tumor dormancy [125]. In small clusters and located scenarios, stochastic mutations and genetic drift may become the most important actors. In these cases, tumor mechanisms to prevent genetic isolation might become relevant in determining the preservation of diversity. For example, tumor cell self-seeding enables a continuous flow of genes from one tumor to another, unifying the allelic frequencies of the different locations, thus creating reservoirs of diversity in the primary tumor or elsewhere, if surgery is not implicated [126,127]. Some authors also defend that horizontal gene transfer could be relevant in this setting [128].

There are other potential benefits related with a reduction of the total heterogeneity. Surgery may prevent the emergence of chemoresistant tumor subclones. In fact, heterogeneity is linked with the selection of pre-existing mutant cells that explain the emergence of primary resistance [129]. The surgery of focal progressions is useful to prevent the expansion of drug resistance in patients with advanced GIST [124]. Finally, if the angiogenic switch is activated, the resection of the primary tumor can change the kinetics of metastases in order to make them more chemosensitive.

The effect of surgery can also be revisited in terms of the cancer stem cell theory. A significant observation is that chemotherapy enriches the content of stem cells in tumors, independently of the response [126]. It is well-known that cancer stem cells are chemoresistant, so debulking surgery could improve the result of chemotherapy by means of eradicating them.

Another exciting finding is the effect orchestrated by the primary tumor to modify the distant soils and facilitate tumor engraftments. Kaplan *et al.* showed that the involved tissues turn more hospitable due to the recruitment of hematopoietic progenitors with angiogenic properties that troop from the bone marrow and form premetastatic niches [130]. Hepatocytes and fibroblasts are also recruited to modify the tumor microenvironment in a similar fashion [131,132]. If we take into account that the consequence of a favorable soil could be significant, the removal of the primary tumor could be a good approach to perturb these networks and prevent the distant control of the areas of engraftment.

## 9. Conclusions

In spite of a current avalanche of data on tumorigenesis, scientists still debate the best unifying model. Surgery has exposed two of its most subtle aspects: distant interactions and variable rhythms of progression. But the crucial questions are not related to the convenience of the evolutionary theory, but to the origin of those rhythms and the influences of multicellularity. The analysis of the Milan and Bloom series implies that tumorigenesis tends to work slowly most of the time, (late relapses have been observed up to 30 years after surgery [133]), but occasional processes such as surgery, inflammation and others, are able to perturb the dynamics of dormant cells, inducing not only a fast-growth but also a rapid phenotypic change. So this topic is analogous to the dispute held over gradualism *versus* punctuated equilibrium [134], in which the current theory of evolution has synthesized both views.

These observations favor a subclone-centered and ecological interpretation of cancer as opposed to non-evolutionary models. The ecological theory clarifies the perturbation of the pre-existing interaction networks, and explains why primary tumors are different from metastasis and why these dissimilarities vanish during the course of tumor progression. It also explains the origin of distant interactions as extensions of a local competition, and the emergence of dormant tumors or aggressive and drug-resistance phenotypes.

It is interesting to notice that the broad mechanisms of evolution that created complex organisms still work within multicellular living beings. The analysis of tumor dormancy has warned against the tendency to adopt a teleological perspective, in which tumorigenesis is just a straight forward process directed towards the maximization of aggressiveness. It is also remarkable that the work of oncological surgeons could argue in favor of punctuated equilibrium as a strong conceptual frame. The aim of further research should be how to protect our patients from the tumorigenic effects of this type of tissular cataclysms.
